# Production of highly bioactive resveratrol analogues pterostilbene and piceatannol in metabolically engineered grapevine cell cultures

**DOI:** 10.1111/pbi.12539

**Published:** 2016-03-07

**Authors:** Ascensión Martínez‐Márquez, Jaime A. Morante‐Carriel, Karla Ramírez‐Estrada, Rosa M. Cusidó, Javier Palazon, Roque Bru‐Martínez

**Affiliations:** ^1^ Plant Proteomics and Functional Genomics GroupDepartment of Agrochemistry and BiochemistryFaculty of ScienceUniversity of AlicanteAlicanteSpain; ^2^ Biotechnology and Molecular Biology GroupQuevedo State Technical UniversityQuevedoEcuador; ^3^ Laboratory of Plant PhysiologyFaculty of PharmacyUniversity of BarcelonaBarcelonaSpain

**Keywords:** grapevine cell culture, metabolic engineering, piceatannol, pterostilbene, resveratrol, *Vitis vinifera*

## Abstract

Grapevine stilbenes, particularly trans‐resveratrol, have a demonstrated pharmacological activity. Other natural stilbenes derived from resveratrol such as pterostilbene or piceatannol, display higher oral bioavailability and bioactivity than the parent compound, but are far less abundant in natural sources. Thus, to efficiently obtain these bioactive resveratrol derivatives, there is a need to develop new bioproduction systems. Grapevine cell cultures are able to produce large amounts of easily recoverable extracellular resveratrol when elicited with methylated cyclodextrins and methyl jasmonate. We devised this system as an interesting starting point of a metabolic engineering‐based strategy to produce resveratrol derivatives using resveratrol‐converting enzymes. Constitutive expression of either *Vitis vinifera* resveratrol O‐methyltransferase (*Vv* ROMT) or human cytochrome P450 hydroxylase 1B1 (*Hs* CYP1B1) led to pterostilbene or piceatannol, respectively, after the engineered cell cultures were treated with the aforementioned elicitors. Functionality of both gene products was first assessed *in planta* by *Nicotiana benthamiana* agroinfiltration assays, in which tobacco cells transiently expressed stilbene synthase and *Vv* ROMT or *Hs* CYP1B1. Grapevine cell cultures transformed with *Vv* ROMT produced pterostilbene, which was detected in both intra‐ and extracellular compartments, at a level of micrograms per litre. Grapevine cell cultures transformed with *Hs* CYP1B1 produced about 20 mg/L culture of piceatannol, displaying a sevenfold increase in relation to wild‐type cultures, and reaching an extracellular distribution of up to 45% of total production. The results obtained demonstrate the feasibility of this novel system for the bioproduction of natural and more bioactive resveratrol derivatives and suggest new ways for the improvement of production yields.

## Introduction

**Figure 1:**
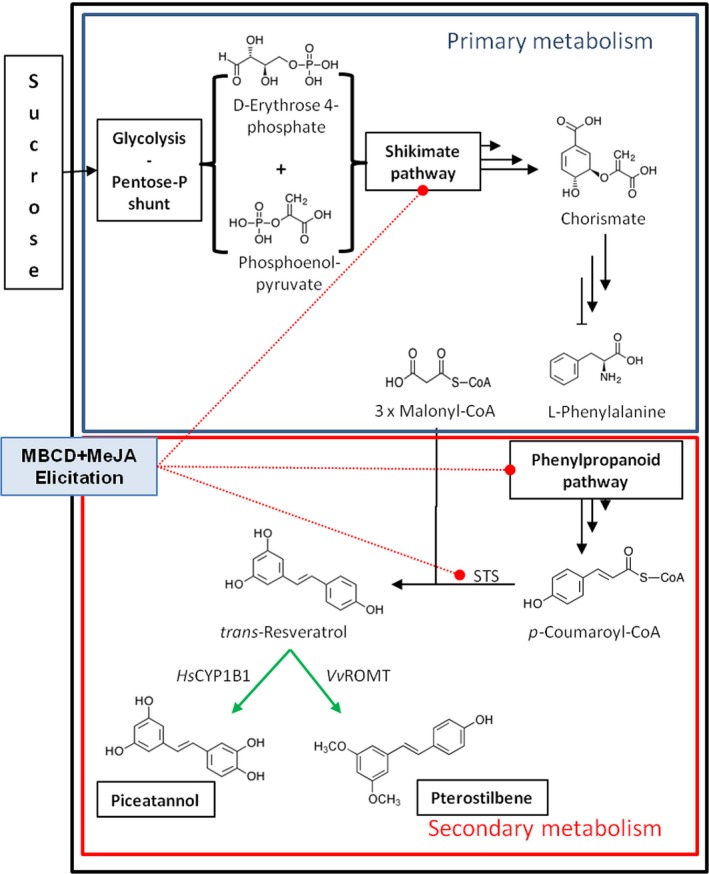
The biosynthetic pathway of stilbene compounds from sucrose as carbon source. The conversion of sucrose carbon into stilbenoids in grapevine cells involves several pathways from primary metabolism, that is glycolysis, pentose phosphate shunt and shikimate, to produce the amino acid phenylalanine as precursor of all polyphenolic compounds, and secondary metabolism, that is phenyl propanoid, to produce 4‐coumaroyl CoA as precursor of stilbenoids (Langcake and Pryce, 1977). The action of STS leads to the production of resveratrol as major stilbenoid, which is strongly induced (red dotted lines) under elicitation conditions with methylated β‐cyclodextrin (MBCD) and methyl jasmonate (MeJA). In transformed grapevine cell lines with either *Vv* ROMT or *Hs* CYP1B1 (green arrows), resveratrol is converted into methylated (pterostilbene) or hydroxylated (piceatannol) derivate, respectively. STS, stilbene synthase; *Vv* ROMT, resveratrol O‐methyltransferase from *Vitis vinifera*;* Hs* CYP1B1, human Cytochrome P450 hydroxylase 1B1.

Stilbenes are a family of polyphenols considered as the most important phytoalexin group in grapevine (*Vitis vinifera*). They derive from the general phenylpropanoid pathway in which *trans*‐resveratrol (3,4′,5‐*trans*‐trihydroxystilbene; *t*‐R) is first formed by stilbene synthases (STS) through the condensation of *p*‐coumaroyl‐CoA with three units of malonyl‐CoA (Figure 1). Free *t*‐R and its glycosylated derivatives are major stilbenes found in leaves and fruit; other stilbenes identified in grapes include piceatannol (*trans*‐3,3′,4,5′‐tetrahydroxy‐stilbene; *t*‐Pn) (Bavaresco *et al*., 2002), while pterostilbene (3′,5′‐dimethoxy‐resveratrol; *t*‐Pt) is produced in small amounts in leaves by a specific resveratrol O‐methyltransferase (*Vv*ROMT) upon microbial challenge (Schmidlin *et al*., 2008).

The bioactivity of *t*‐R has been widely studied and reviewed, and includes the prevention or slowing of a wide variety of illnesses, including cancer, obesity and cardiovascular diseases, as well as the extension of the lifespan of various organisms (Aggarwal *et al*., 2004; Baur and Sinclair, 2006; Das and Das, 2007; Fulda, 2010; Pervaiz and Holme, 2009). Although these beneficial properties have been broadly demonstrated *in vitro*, transfer to *in vivo* systems is restricted by the limited oral bioavailability of *t*‐R (Asensi *et al*., 2002). Some *t*‐R analogues such as polyhydroxy and polymethoxy derivatives exhibit higher pharmacological activity than the parent compound (Szekeres *et al*., 2011). The naturally occurring *t*‐Pt, 3,4′,5‐trimethoxystilbene, pinostilbene (3,4′‐dihydroxy‐5‐methoxy‐trans‐stilbene) and desoxyrhapontigenin (3,5‐dihydroxy‐4′‐methoxy‐trans‐stilbene) are found in some plants but at a very low concentration (Wang *et al*., 2010). Their bioavailability is five to ten times higher than that of *t*‐R (Lin *et al*., 2010; Remsberg *et al*., 2008), and their anticancer and cancer chemopreventive activity have been demonstrated in both model cell culture systems and solid tumours (Pan *et al*., 2009; Suh *et al*., 2007). The efficacy of orally administered *t*‐R depends on its absorption, metabolism and tissue distribution (Yu *et al*., 2006). In humans, hydroxy resveratrol derivatives such as *t*‐Pn, produced as a major metabolite of resveratrol by human liver CYP1B1 and CYP1A2 enzymes (Piver *et al*., 2004; Potter *et al*., 2002), have revealed additional functions to anticancer properties. *t*‐Pn obtained from a plant extract was identified as an antileukaemic principle (Ferrigni *et al*., 1984), displaying inhibition of tyrosine kinases (Geahlen and McLaughlin, 1989) involved in cell proliferation (Fleming *et al*., 1995), tubulin phosphorylation (Peters *et al*., 1996), the phosphorylation of DNA transcription factors (Su and David, 2000), different types of cancer (Thakkar *et al*., 1993) and antiparasitic activity (Piotrowska *et al*., 2012).

The therapeutic value of *t*‐R and its derivatives has stimulated the demand for nutraceutical, cosmetic and pharmaceutical uses. Sources of resveratrol include extraction from plant raw material, chemical synthesis and bio‐production, the latter having a number of advantages that combine cost‐effectiveness with being environmentally friendly. The bioproduction of *t*‐R (reviewed in Donnez *et al*., 2009) by grapevine cell cultures elicited with cyclodextrins (CD) alone (Bru *et al*., 2006) or combined with methyl jasmonate (MeJA) (Lijavetzky *et al*., 2008; Martinez‐Esteso *et al*., 2009) has proven to be the most efficient system, with a 30% overall conversion of the sugar carbon into resveratrol carbon (Almagro *et al*., 2013). Metabolic engineering of microorganisms is also being intensively investigated as an alternative way for the bioproduction of *t*‐R and analogues (Jeong *et al*., 2014; Kim *et al*., 2009; Li *et al*., 2015; Lim *et al*., 2011; Wang *et al*., 2010), but the yield of elicited grapevine cell cultures is still unbeaten.

This motivated us to investigate the production of the hydroxylated and methylated derivatives of *t*‐R in grapevine cell suspensions. *t*‐Pn accumulates in grape berries as a constitutive stilbene (Bavaresco *et al*., 2002) and *t*‐Pt in grapevine leaves infected by *Plasmopara viticola* (Langcake *et al*., 1979; Schmidlin *et al*., 2008); low amounts of pterostilbene are also produced during the preparation of grapevine protoplasts (Commun *et al*., 2003). This compound was also found in healthy grape berries as a constitutive stilbene (Pezet and Pont, 1988), but not in grapevine cell cultures, even after elicitation (Bru *et al*., 2006; Lijavetzky *et al*., 2008; Martinez‐Esteso *et al*., 2011a). To investigate if elicited cell cultures could become a new source of *t*‐Pt and higher levels of *t*‐Pn, we developed transgenic grapevine cell lines constitutively expressing *Hs*CYP1B1 or *Vv*ROMT, and carried out elicitation experiments to supply *t*‐R substrate for its conversion into the hydroxylated *t*‐Pn or the methylated *t*‐Pt, respectively (Figure 1). As a result, a number of cell lines integrating the transgene expressed the corresponding protein and were able to accumulate *t*‐Pn or *t*‐Pt upon methylated β‐cyclodextrin (MBCD) and MeJA elicitation. Therefore, making use of enhanced metabolic capacity of grapevine cells upon elicitation to synthesize *t*‐R, we have designed a metabolic engineering strategy to obtain *t*‐Pt and *t*‐Pn as a way to diversify the stilbene‐producing metabolic capacities of grapevine cell cultures.

## Results

### Biosynthesis of resveratrol derivatives in *Nicotiana* leaves

**Figure 2:**
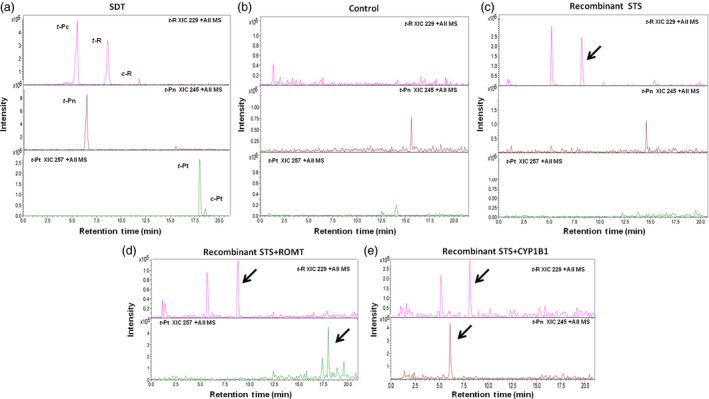
Production of t‐Pn and t‐Pt in planta by agroinfiltration. ROMT and CYP1B1 activities were assessed *in planta* using *Nicotiana benthamiana* agro‐infiltration. The tobacco leaves were excised six days after agro‐infiltration and extracts analysed by HPLC‐ESI‐MS. Extracted ion chromatograms from resveratrol (XIC 229), piceatannol (XIC 245) and pterostilbene (XIC 257) are shown. (a) Standards. (b) Control, leaf extract agroinfiltrated with rolD‐EgfpER construct (Martínez‐Márquez *et al*., 2015). (c) Leaf extract agroinfiltrated with STS inducible by MBCD+MeJA. (d) Leaf extract agroinfiltrated with ROMT and STS inducible by MBCD+MeJA. (e) Leaf extract agroinfiltrated with CYP1B1 and STS inducible by MBCD+MeJA. Peaks: *t*‐Pc, *trans*‐piceid; *t*‐R, *trans*‐resveratrol; *t*‐Pn, *trans*‐piceatannol; *t*‐Pt, *trans*‐pterostilbene, small amounts of *cis*‐resveratrol (*c*‐R) and *cis*‐pterostilbene (*c‐*Pt) isomers are also present. Identity of peaks was confirmed by HPLC‐ESI‐MS/MS analysis (data not shown). Note that *t*‐Pc can be detected as a molecular ion [M+H^+^]=229 due to extensive in‐source fragmentation of this compound.

*Agrobacterium‐*mediated transient transformation of *Nicotiana* was used to assess the functionality of the cloned genes ROMT and CYP1B1. As *Nicotiana* lacks the stilbenoid pathway, an STS gene was simultaneously transiently expressed to supply ROMT or CYP1B1 with *t*‐R substrate *in planta*. In previous studies, several STS isoforms were shown to be induced in *V. vinifera* cv. Gamay and cv. Monastrell cell culture elicited with MBCD and MeJA (Martinez‐Esteso *et al*., 2011a). Some of these isoforms such as Vst1 (Melchior and Kindl, 1991) are known to produce *t*‐R when introduced as a transgene into a range of species including tobacco (*Nicotiana* spp.), tomato (*Solanum lycopersicum*), papaya (*Carica papaya*) and grapevine (Coutos‐Thévenot *et al*., 2001; Hain *et al*., 1990; Hipskind and Paiva, 2000; Thomzik *et al*., 1997). Instead, we selected an STS gene (Acc. XM_002264953) not previously characterized whose protein product accumulates in response to MBCD and MeJA elicitors in grapevine cell cultures, to be coexpressed with ROMT or CYP1B1. As a control, tobacco leaves were infiltrated with *Agrobacterium* harbouring a rolD‐EgfpER construct (Martínez‐Márquez *et al*., 2015). No stilbenes were detected in extracts from leaves expressing GFP (Figure 2b). Significant amounts of *t*‐R and its glycosylated form piceid (*t*‐Pc) accumulated when STS alone was transiently expressed (Figure 2c) while the coexpression of STS and ROMT resulted in an accumulation of *t*‐Pt together with a decrease in the *t*‐R and *t*‐Pc peaks (Figure 2d) and the coexpression of STS and CYP1B1 resulted in a decrease in the resveratrol peak, together with an accumulation of *t*‐Pn (Figure 2e).

### Establishment of grapevine transformed cell culture

*A. tumefaciens* strain EHA 105 harbouring pJCV52‐ROMT and *A. tumefaciens* strain C58C1 (pGV2260) harbouring pK7WG2D‐CYP1B1 successfully transformed callus of *Vitis,* from either Gamay or Monastrell varieties. After 4 weeks on selection medium, paramomycin‐resistant callus colonies were large enough to harvest the biomass and transfer to new plates with fresh selection medium. In selection medium, the nontransformed material did not grow and turned brown while transformed callus grew vigorously. Within 3–4 months of initial transformation, sufficient callus material was obtained to launch liquid cultures, thus rapidly growing cell suspensions were established. Transgenic suspension cultures growing in paramomycin‐containing medium showed no detectable difference in cell growth when compared to the wild parent cell line grown in paramomycin‐free medium. *Vitis* transgenic cultures have been maintained under continuous paramomycin selection for more than 7 months and other 4 months in paramomycin free with no loss in vigour. On average, 10 and 17 transformed calli were obtained per 1 g FW of plated *Vitis* Monastrell and Gamay varieties cells, respectively, with no differences between the source material, either cell suspension or callus. About 60–65% of *Vitis* transformed calli could be successfully maintained under continuous paramomycin selection.

### Molecular characterization of grapevine transformed cell culture

**Figure 3:**
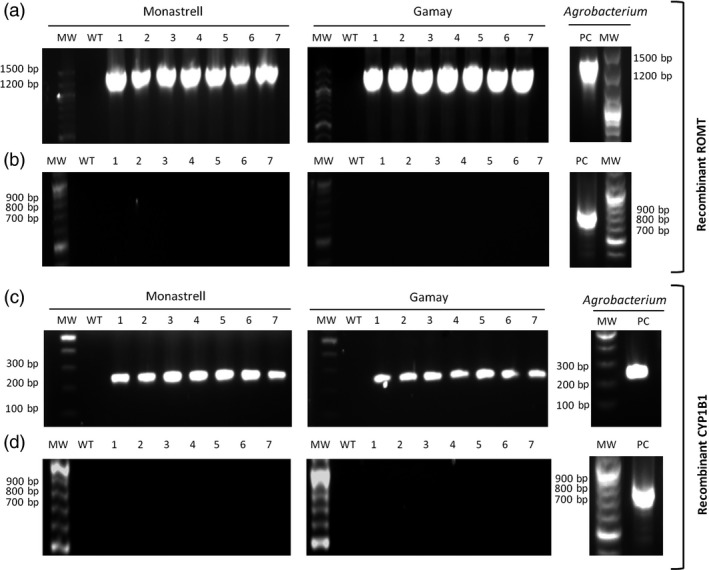
PCR amplification products from genomic DNA of transgenic callus lines of *Vitis vinifera* Monastrell and Gamay varieties. Amplification was carried out using P35S‐ROMT, CYP1B1 and virB specific primers (Table S3). (a,c) Amplification products of P35S‐ROMT (a) and CYP1B1 (c) from transgenic lines of *V. vinifera* Monastrell and Gamay varieties. (b, d) Amplification products of virB from transgenic lines of *V. vinifera* Monastrell and Gamay varieties. WT. wild‐type callus negative control using nontransgenic genomic DNA of *Vitis* cells, 1–7 randomly selected transgenic callus. PC: positive controls using plasmidic DNA as template.

Seven randomly selected grapevine transgenic calli, as well as the control wild‐type calli, were checked for plant genome T‐DNA integration of ROMT and CYP1B1 genes by PCR amplification using primer pairs specific for ROMT and CYP1B1 under control of P35S, and for eventual *Agrobacterium* contamination using virB primer pairs; ROMT and CYP1B1 were present in all of their respective transgenic clones, but not in the wild type (Figures 3a and 3c), whereas the virB PCR product was absent in all transgenic lines (Figures 3b and 3d). These results proved that the transgenic cultures were actually transformed with either ROMT or CYP1B1 and were not contaminated with *Agrobacterium*.

**Figure 4:**
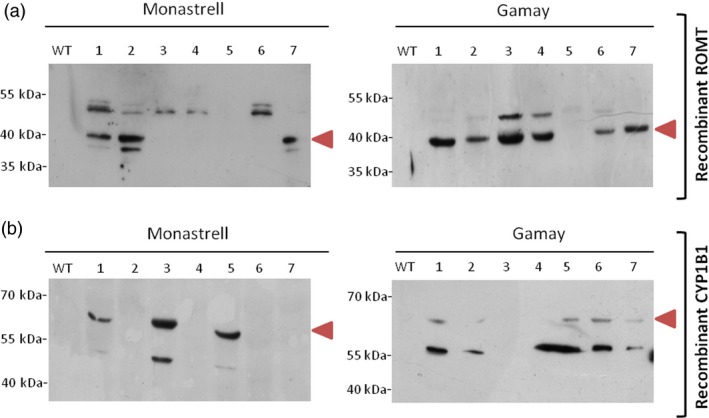
Expression of ROMT and CYP1B1 recombinant protein in cell cultures of *Vitis vinifera* Monastrell and Gamay varieties. (a) Expression of HA‐tag fusion proteins was confirmed by Western blot analysis with anti‐HA‐tag antibody. (b) Expression of human liver CYP1B1 protein was confirmed by Western blot analysis with CYP1B1 antibody. WT, wild‐type callus negative control using protein extract of nontransformed *Vitis* cells, 1–7 randomly selected transgenic callus.

Immunodetection of HA‐tagged ROMT protein and CYP1B1 protein in the soluble fraction of the corresponding transformed *Vitis* cultures was used to assess for recombinant protein synthesis in transgenic calli, as well as the negative control wild‐type callus. We were able to visualize HA‐tagged ROMT protein bands in Western blots with anti‐HA‐tag antibody. A HA‐tagged ROMT band of the expected theoretical molecular weight of 41.7 kDa was clearly distinguished in the soluble protein fraction of *Vitis* transformants callus of Gamay (6 of 7) and Monastrell (3 of 7), while it was absent in nontransformed cultures (Figure 4a). Furthermore, an additional band of approximately 50 kDa was detected in 2 and 5 transformant calli of Gamay and Monastrell, respectively, and a band of apparent Mw of 37 kDa was also detected in two Monastrell calli where the 41.7 kDa band was present as well. CYP1B1 protein bands in Western blots were visualized with anti‐CYP1B1 antibody. A CYP1B1 band was clearly distinguished in the soluble protein fraction (Figure 4b) of all *Vitis* transformants callus corresponding to their theoretical molecular weight of 60.8 kDa while it was absent in nontransformed cultures. A second band of ca. 55 kDa appeared which most likely corresponded to a *Hs*CYP1B1 degradation product, and according to the band intensity, degradation extent is higher in Gamay than in Monastrell.

**Figure 5:**
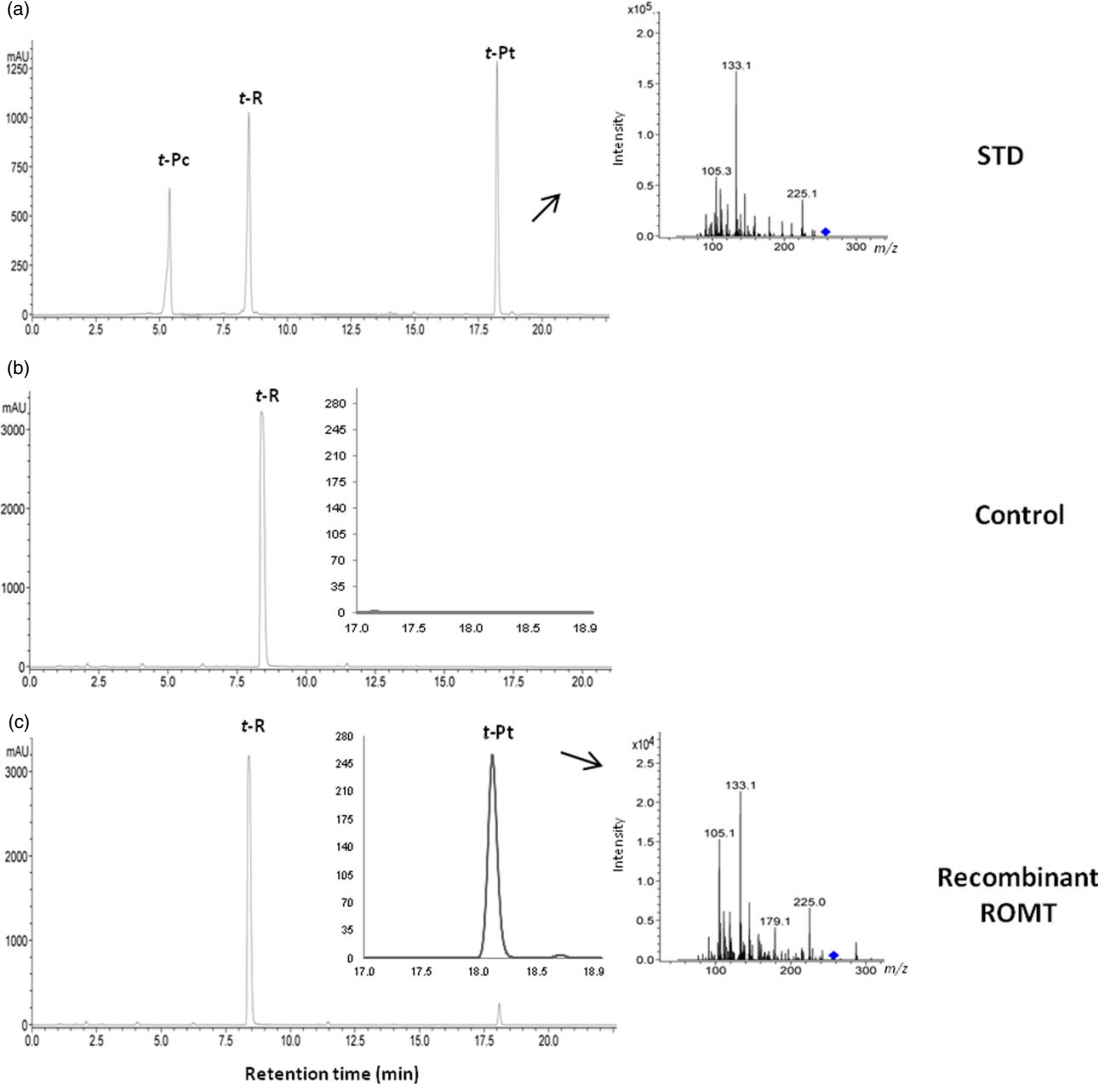
Conversion of *t‐*R into *t*‐Pt by cell extract of *Vv* ROMT‐transformed grapevine cell cultures. HPLC‐ESI‐MS analysis of reaction products following incubation of recombinant HA‐tagged ROMT with 500 μm resveratrol in the presence of SAM (25 mm). (a) Standards. Reaction was carried out with 400 μg total soluble protein of either wild (b) or transgenic callus cv. Monastrell (c) and was allowed to proceed for 24 h. A chromatographic profile is shown, and identity of peaks was confirmed by HPLC‐ESI‐MS/MS analysis. Peak *t*‐Pc, *trans*‐piceid; Peak *t*‐R, *trans*‐resveratrol; Peak *t*‐Pt, *trans*‐pterostilbene.

To characterize recombinant ROMT activity, total soluble protein extract of grapevine transformant and wild‐type callus was incubated with resveratrol and SAM. HPLC‐MS analysis of reaction products after 24 h incubation with grapevine transgenic protein extract showed two peaks corresponding to resveratrol and pterostilbene (Figure 5). In the protein extract of wild‐type callus, there was revealed a single peak corresponding to resveratrol, thus providing solid evidence of the functionality of ROMT in transgenic grapevine lines.

### Accumulation of stilbenoids in grapevine cell cultures upon elicitation

It is well known that elicitation treatments of *V. vinifera* cv. Gamay and cv. Monastrell cell suspensions with MBCD combined with MeJA lead to large increases in accumulation of *t*‐R (Lijavetzky *et al*., 2008) because *t*‐R is abundantly synthesized in treated cells and continuously translocated to the medium. However, the treatment with MBCD and MeJA does not lead to *de novo* synthesis of *cis*‐resveratrol (*c*‐R), being a minority metabolite as compared to *t*‐R (Martinez‐Esteso *et al*., 2011a). Inside the cells, *trans*‐piceid (*t*‐Pc) was already present in nontreated cells and underwent little or no significant change in abundance in response to the treatment, results that correlate well with our present study. *t*‐Pn in elicitation treatments of *Vitis* cv. Gamay and cv. Monastrell cell suspensions with MBCD+MeJA is synthesized in low amounts, being a minority metabolite as compared to *t*‐R, while *t*‐Pt has not been reported so far.

**Figure 6:**
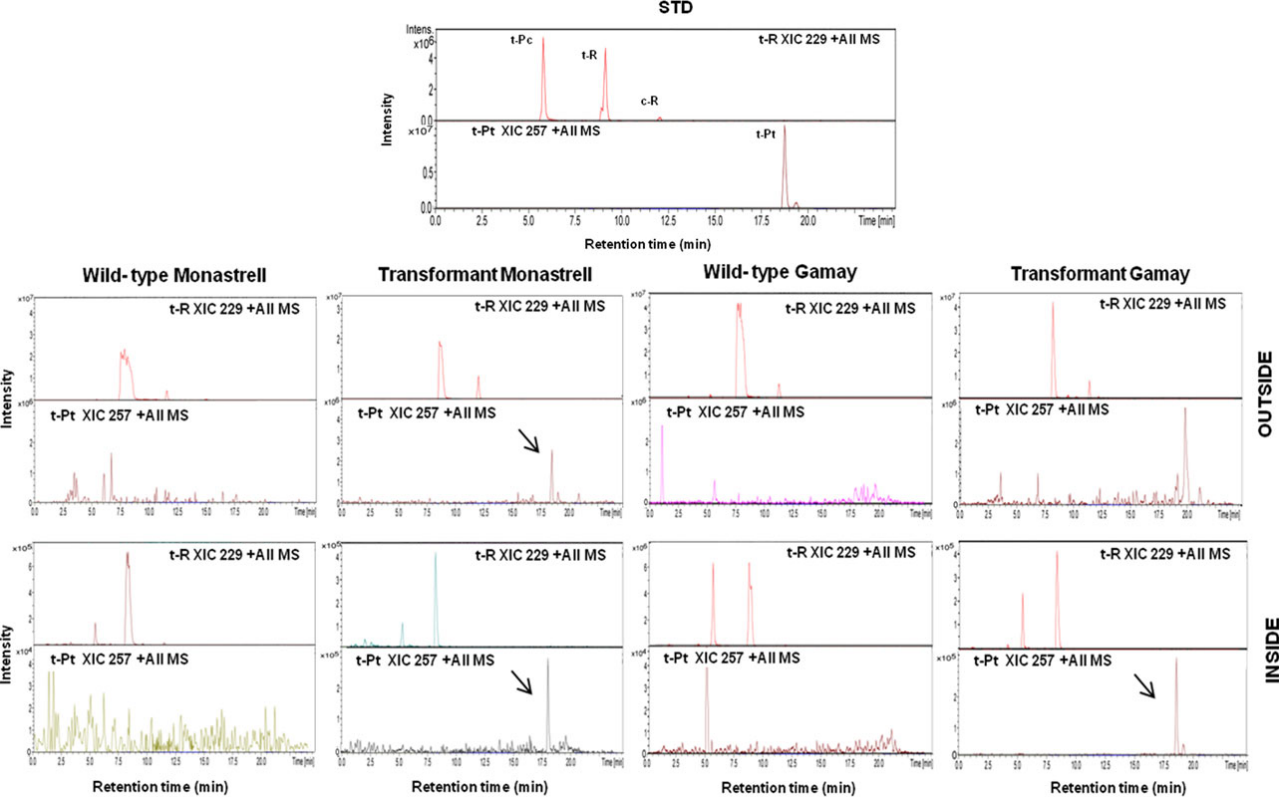
Production of *t*‐Pt by elicited VvROMT‐transformed grapevine cell cultures. Stilbenoid HPLC‐ESI‐MS analysis of transformed *Vitis* cv. Monastrell and cv. Gamay cell extracts and extracellular media. Stilbenes content was determined in extracts of transgenic and wild‐type cell cultures at 168 h. Extracted ion chromatograms from pterostilbene (XIC 257) and resveratrol (XIC 229) are shown, and identity of peaks was confirmed by MS/MS the spectra (See Figure S1 and S2 for MS/MS spectrum of pterostilbene in *Vitis* cv. Monastrell and cv. Gamay cell extracts and extracellular medium). Peaks: t‐Pc, *trans*‐piceid; t‐R, *trans*‐resveratrol; t‐Pt, *trans*‐pterostilbene.

Once suspension cultures of ROMT‐expressing transgenic *Vitis,* cells were established *t‐*R and *t*‐Pt productivity upon MBCD+MeJA treatment was determined. Figure 6 shows the presence of the stilbenoids accumulated after 168 h of incubation with MBCD+MeJA determined by HPLC‐ESI‐MS. These analyses include *t*‐R, glycosylated form of resveratrol, *t*‐Pc and *t*‐Pt. The *t*‐Pt was found both inside and outside the transgenic culture of Monastrell and inside the transgenic culture of Gamay. Elicitation of transgenic culture resulted in a decrease in the *t*‐R, together with an accumulation of *t*‐Pt, demonstrating that the *t*‐R had been modified by double regiospecific dimethoxylation. The *t*‐Pt accumulation in the Monastrell cells culture was higher than in the Gamay ones.

**Figure 7:**
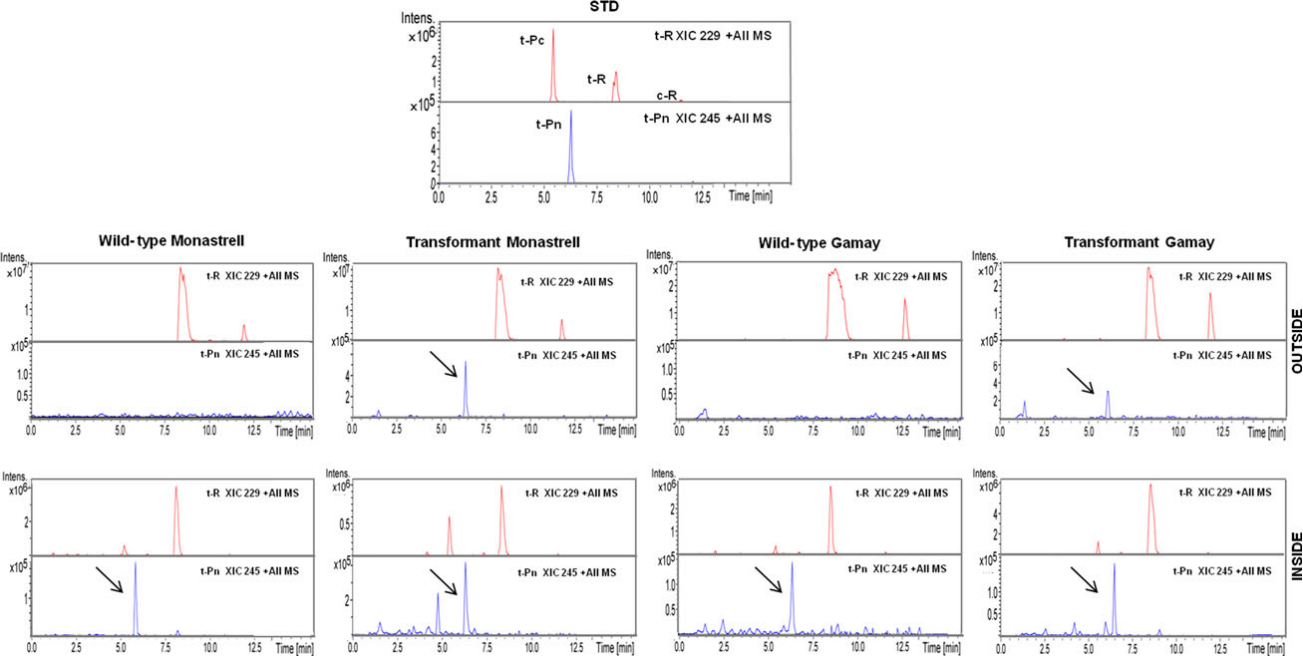
Production of *t*‐Pn by elicited HsCYP1B1‐transformed grapevine cell cultures. HPLC‐ESI‐MS analysis of *Vitis* cv. Monastrell and cv. Gamay cell extracts and extracellular media. Content of stilbenes was determined in extracts of transgenic and wild‐type cell cultures at 72 h. Extracted ion chromatograms from piceatannol (XIC 245) and resveratrol (XIC 229) are shown, and identity of peaks was confirmed by the MS/MS spectra (See Figure S3 and S4 for MS/MS spectrum of piceatannol in *Vitis* cv. Monastrell and cv. Gamay cell extracts and extracellular medium). Peaks: t‐Pc, *trans*‐piceid; t‐R, *trans*‐resveratrol; t‐Pn, *trans*‐piceatannol.

After suspension cultures of CYP1B1‐expressing transgenic *Vitis,* cells were established *t‐*R and *t*‐Pn productivity was determined. Figure 7 shows the presence of the stilbenoids accumulated after 72 h of incubation with MBCD+MeJA determined by HPLC‐ESI‐MS. The *t*‐Pn was found in both transgenic and wild‐type cell suspensions, being the accumulation in the transgenic cell suspensions higher than in wild‐type cell suspensions. Elicitation of transgenic culture resulted in a decrease of the total *t*‐R, together with an increase of the total *t*‐Pn accumulation. This result suggested that the *t*‐R had been modified by regiospecific hydroxylation. The accumulation in the transformant Monastrell culture was higher than in the Gamay one.

**Figure 8:**
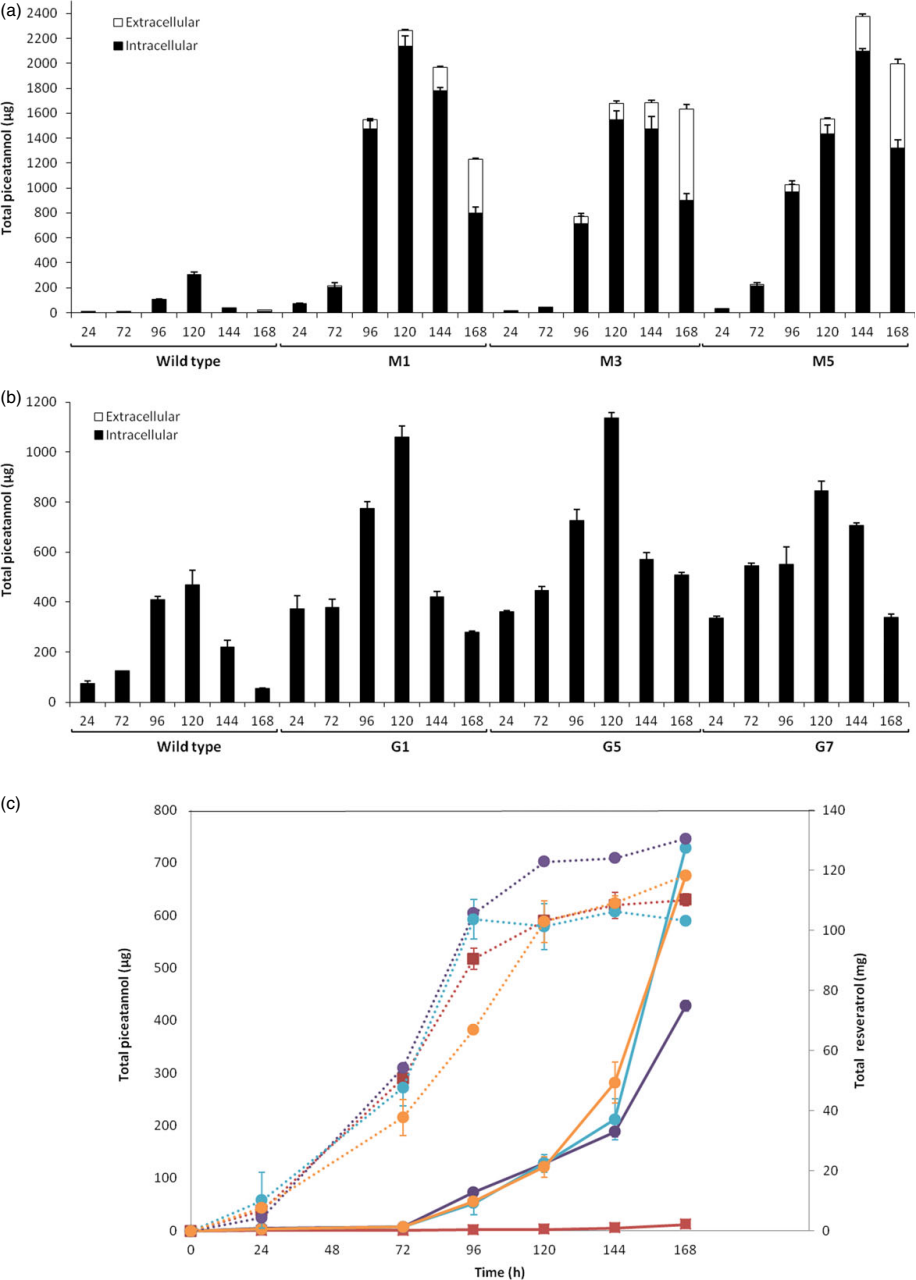
Time‐course accumulation of *t*‐Pn in elicited HsCYP1B1‐transformed grapevine cell lines. Total piceatannol production in *Vitis* cv. Monastrell (a), cv. Gamay (b) and extracellular accumulation of resveratrol and piceatannol in cv. Monastrell (c) cell culture elicited with 50 mm MBCD and 0.1 mm MeJA between 24 and 168 h of incubation. Twenty grams of cells was suspended in sterile fresh standard medium containing elicitors brought to a final 100 mL. Total piceatannol amounts are calculated per 100 mL of cell culture. In (a) and (b), black bars: intracellular content; white bars: extracellular content. In (c), each Monastrell cell line is represented with a different colour; solid lines: piceatannol, scaled to the left axis; dotted lines: resveratrol, scaled to the right axis; wild type: solid squares; transformed lines: solid circles. M. Monastrell transformed lines; G: Gamay transformed lines. Data are the mean of three independent biological replicates ± SD.

The total accumulation of *t‐*Pn between 24 and 168 h after elicitation was analysed by HPLC‐ESI‐MS (Figure 8). Maximal level, both for wild type and almost all transformed lines, was achieved at 120 h. Transgenic cell suspensions of Monastrell and Gamay varities produced about 2 and 1 mg per 100 mL culture, respectively, displaying up to a 7‐ and 2.5‐fold increase in relation to respective wild‐type cultures. A significant amount of *t*‐Pn was obtained from extracellular medium of the Monastrell while only traces were detected in the Gamay cells. The maximum accumulation occurred in the media at the end of the experiment, at 168 h, representing between 34% and 45% of the total *t*‐Pn production by Monastrell cells while in case of the Gamay cells the extracellular content of *t*‐Pn represented only a 0.05% of the total *t*‐Pn, at best. When extracellular *t*‐Pn was compared with *t*‐R in Monastrell (Figure 8c), an interesting behaviour was observed. The *t*‐R increased exponentially from the beginning of elicitation, decelerating after 96 h and reaching an accumulation plateau after 120 h; on its side, *t*‐Pn displayed a lagged accumulation of 72 h, increased almost linearly up to 144 h and then fast between 144 and 168 h, when the last sample was measured. There is an apparent correlation between the moment of resveratrol deceleration and the delayed start of extracellular *t*‐Pn accumulation. This result suggests a competence between resveratrol and piceatannol for the transport system to the extracellular medium and a higher specificity of the mechanism for resveratrol.

## Discussion

The chemopreventive, therapeutic and cosmetic properties of resveratrol and its methoxylated and poly‐hydroxylated derivatives, as well as their scarcity in natural plant sources, have prompted considerable efforts to engineer plant and microbial organisms for the production of these compounds (Delaunois *et al*., 2009; Donnez *et al*., 2009). Increased bioavailability and bioactivity support the interest in these types of *t*‐R derivatives, in particular *t*‐Pt and *t*‐Pn (Lin *et al*., 2010; Piotrowska *et al*., 2012; Remsberg *et al*., 2008; Szekeres *et al*., 2011).

From an economical perspective, these compounds should ideally be obtained as the fermentation end‐products from a cheap carbon source, that is sugars, rather than as bioconversion products of metabolically related precursors. Extracellular accumulation is another desirable feature, allowing the product to be recovered more easily and in a purer state. Metabolically engineered microorganisms, mainly *E. coli* and yeast, plant cell cultures and whole plants, are the type of biological systems used for such purposes (Donnez *et al*., 2009).

*Escherichia coli* has been engineered to produce *t*‐Pt from tyrosine in a four‐step pathway, the intermediates being *p*‐coumaric acid, *p*‐coumaroyl‐CoA and resveratrol (Kang *et al*., 2014). The doubly methylated target product *t*‐Pt was formed when resveratrol was used as a precursor but not as a fermentation product of the carbon source, with only *p*‐coumaric acid, resveratrol and mono‐methylated derivatives being detected. *Nicotiana* and *Arabidopsis* plants stably transformed with peanut stilbene synthase‐3 and sorghum O‐methyl transferase‐3 were found to accumulate *t*‐Pt *in planta* (Rimando *et al*., 2012). However, although these plants may benefit from higher antifungal properties of this compound, their use as a *t*‐Pt source would involve a complex downstream extraction and purification of the final product.

The bio‐production of *t*‐Pn is currently much less developed than that of *t*‐Pt, and only enzymatic bioconversion of resveratrol has been reported, using human liver microsomes (Piver *et al*., 2004; Potter *et al*., 2002), bacterial cytochrome P450 BM3 and its mutants (Kim *et al*., 2009) and bacterial tyrosinase (Lee *et al*., 2012, 2015). No studies towards a metabolic engineering production strategy have been reported. As the most productive *t*‐R system to date is an elicited grapevine cell culture (Almagro *et al*., 2013; Donnez *et al*., 2009), the target compound accumulating mainly in the extracellular medium (Almagro *et al*., 2013; Donnez *et al*., 2009), we considered this as an interesting starting point for the production of *t*‐Pt and *t*‐Pn in metabolically engineered cell lines.

The designed strategy consisted of using grapevine cell lines in which the endogenous biosynthetic pathways leading from sucrose to *t*‐R would be extended to produce the target derivatives by the constitutive expression of either a methyl transferase or a cytochrome P450‐dependent hydroxylase able to act on *t*‐R (Figure 1). The treatment of the engineered lines with elicitors would supply large amounts of *t*‐R to the recombinant enzymes and eventually lead to the extracellular accumulation of their reaction products.

Suitable enzymes were selected on the basis of literature reports, a major issue being reaction regiospecificity, so as to obtain only the desired single product, that is either *t*‐Pt or *t*‐Pn. Among methyl transferases, we selected grape *Vv*ROMT (Schmidlin *et al*., 2008) as it could be expected to exhibit higher specificity. The cytP450 was selected as the CYP1B1, due to its eukaryotic origin and its importance as a tumour suppressor, although both 1B1 and 1A2 isoenzymes produce a second tetrahydroxystilbene product, not structurally well characterized, named M1 (Piver *et al*., 2004).

The functionality of *Vv*ROMT and *Hs*CYP1B1 was tested first *in planta*, for the first time in case of the hydroxylase, and rendered the expected products, *t*‐Pt and *t*‐Pn, respectively, in co‐agroinfiltration experiments with a *Vv*STS in *Nicotiana* (Figure 2d, e). The functionality of *Vv*ROMT expressed in grapevine cell cultures was additionally confirmed in an *in vitro* enzymatic assay. Highly efficient stable *Agrobacterium*‐mediated transformation of somatic grapevine cells recently developed in our laboratory (Martínez‐Márquez *et al*., 2015) was used to establish grapevine callus cell lines transformed with either *Vv*ROMT or *Hs*CYP1B1. Characterization of the cell lines for transgene integration was positive in all the lines growing in the selection medium but not all of them produced the recombinant protein (Figures 3 and 4). Inconsistency between gene, transcript and protein presence, even under the control of a constitutive promoter such as pCaMV35S, has been reported previously, the phenomenon being attributed to fluctuations in environmental conditions and stress, which may affect gene expression, transcript steady‐state levels or transgene silencing (Boyko *et al*., 2010; Kotakis *et al*., 2010). In the case of ROMT, immune‐reactive bands were often observed with Mw significantly higher than expected and it was also associated with no detection of the *t*‐Pt product in subsequent metabolite analysis. We attributed this failure to a possible fast turnover of the ROMT protein, likely mediated by ubiquitination, which would increase the Mw as observed in Western blot analysis. In summary, despite the occurrence of a number of unproductive transformation events, a remarkable number of cell lines were selected for *t*‐Pt and *t*‐Pn production assays.

The characterization of transformed cell lines involved the establishment of liquid cell suspension cultures, followed by elicitation with MBCD+MeJA and analysis of stilbene compounds accumulated both inside cells and in the extracellular medium. Grapevine cells respond to elicitation with MBCD+MeJA increasing the abundance of several STS protein isoforms (Martinez‐Esteso *et al*., 2011a), which correlates with the large accumulation of *t*‐R both in the cells and particularly the extracellular medium (Lijavetzky *et al*., 2008; Martinez‐Esteso *et al*., 2011a). In transformed cell lines, this would also lead to resveratrol and eventually to resveratrol derivative synthesis. Elicitation of transgenic cultures resulted in the accumulation of either *t*‐Pt (Figure 6; Figure S1 and S2) or *t*‐Pn (Figure 7; Figure S3 and S4), thus demonstrating the feasibility of the designed strategy to produce resveratrol derivatives. Their precursor *t*‐R was also found but at a lower level than in the wild‐type culture, in accordance with its role as an intermediate in the synthesis of the final derivative products.

In previous work, we ascertained that transformation of grapevine cells itself does not affect the level of resveratrol production upon elicitation (Martínez‐Márquez *et al*., 2015). Overall, the production of *t*‐Pt per 100 mL of culture (low microgram range) was much lower than that of *t*‐Pn (low milligram range), which in turn was modest in relation to the overall production of resveratrol (100 mg range). A likely reason for this unanticipated low rate of conversion of resveratrol into its derivatives may be a strongly competing (still unknown) resveratrol transport system to the extracellular medium. Another possible explanation is that in addition to being a substrate, if present in a high intracellular concentration, resveratrol can act as an inhibitor of CYP1B1 (Chang *et al*., 2000), which may play against *t*‐Pn production (Chang *et al*., 2000). Another factor could be a fast turnover of the recombinant protein, either ROMT or CYP1B1, suggested by the presence of additional immunoreactive bands, especially in the case of ROMT, and finally, end‐product stability might also contribute to reducing the product accumulation.

The inducibility of the *Vv*ROMT gene by biotic‐stress (Schmidlin *et al*., 2008) can lead to a fast turnover (Li *et al*., 2012) through specific mechanisms, which might be operating fully upon its homologous overexpression in grapevine. In addition, we observed that *t*‐Pt disappeared when added to either wild‐type or transgenic grapevine cultures. Five‐millimolar MBCD slowed down that process (results not shown) and this point to low stability of *t*‐Pt in the culture medium. The combination of these mechanisms may be a major cause of the limited accumulation of *t*‐Pt, and thus, alternative strategies should be designed which may include the use of alternative OMTs and the efficient removal of the product during the cell culturing.

While *t*‐Pt was detected only in elicited ROMT‐transformed grapevine cells, *t*‐Pn was present in small amounts in the wild‐type culture, with a maximum accumulation at 120 h, thus indicating of some endogenous, still uncharacterized, resveratrol hydroxylase activities. In CYP1B1‐transformed cultures, *t*‐Pn accumulation increased in all tested lines of both varieties, thus providing evidence for the activity of the transgene. Both resveratrol derivatives were found both inside and outside cells, with greater abundance in the Monastrell cells which were more productive overall than the Gamay ones. In fact, *t*‐Pt presence in the extracellular medium could not be confirmed in the latter. The low accumulation of *t*‐Pt precluded its reliable quantification, but in the case of *t*‐Pn the percentage of extracellular production in Monastrell increased rapidly after 144 h, reaching up to 45% at 168 h, while extracellular *t*‐Pn in Gamay was only 0.05% at best. Extracellular resveratrol was the most abundant form, in agreement with previous reports (Lijavetzky *et al*., 2008; Martinez‐Esteso *et al*., 2011a). This result suggests that the mechanisms of stilbenoid transport to the extracellular medium operate less efficiently for *t*‐Pn/*t*‐Pt than for resveratrol and differ between grapevine varieties. In addition, the delayed start of extracellular accumulation of *t*‐Pn and the moment of deceleration of resveratrol accumulation correlate quite well, suggesting that the transport system displays a higher specificity for resveratrol and *t*‐Pn is moved out only when resveratrol transport ceases. Nothing is known about such a mechanism, but the results obtained here point to transporter proteins that display stringent substrate specificity, rather than vesicle‐mediated secretion whose cargo‐type might be less specific.

Another differential effect between the grapevine varieties concerns to *t*‐Pn production enhancement in transgenic lines, which was higher in Monastrell. If we consider that Gamay, but not Monastrell, constitutively accumulates the resveratrol glycosylated derivative piceid (Martinez‐Esteso *et al*., 2011a), a reason for the less efficient conversion of *t*‐R into *t*‐Pn in the Gamay cells may be competition of the cytochrome P450 hydroxylase with glycosyl transferases for resveratrol (Hall and De Luca, 2007), in addition to the competing extracellular transport mechanisms. All the results presented here provide new information about stilbenoid metabolism in grapevine cell cultures and are currently being applied to re‐shape our strategy to overcome the potential hurdles that hinder an efficient production of *t*‐Pn in transformed grapevine cells.

### Concluding remarks

Here we have demonstrated the feasibility of a metabolic engineering strategy in grapevine cells to produce the resveratrol derivatives *t*‐Pt and *t*‐Pn, endowed with enhanced pharmacological and pharmacodynamical properties. Use of alternative methylating enzymes and end‐product stabilization could greatly improve the yields of *t*‐Pt production. The engineered production of *t*‐Pn has been carried out for the first time, both *in planta* and in cell cultures, and the results obtained point promisingly towards a future improved strategy to enable an economically viable bioproductive system.

## Material and methods

### Plant material

*Vitis vinifera* L. cv. Gamay callus was kindly supplied by Drs. J. C. Pech and A. Latché (ENSA, Toulouse, France) in 1989. *Vitis vinifera* L. cv. Monastrell albino calli were established in 1995 as previously described (Zapata *et al*., 1995). These cell lines were maintained in both solid and liquid cultures in Gamborg B5 medium (Duchefa, Haarlem, The Netherlands) by periodical subcultures as described elsewhere (Bru *et al*., 2006).

*Vitis vinifera* L. cv Aledo leaves were obtained from a commercial plot of table grapes located in Agost, Alicante, Spain, in June 2011 and transported to the laboratory at 4 °C. *Nicotiana benthamiana* plants were obtained from seeds germinated and grown on potting soil in a greenhouse at a temperature of 25 °C, with 16 h light/8 h dark photoperiod, until they were 3–5 weeks old.

### Reagents

Except if indicated between brackets chemicals and kits were purchased from Sigma‐Aldrich (Madrid, Spain), including methylated β‐cyclodextrin (MBCD) and methyl jasmonate (MeJA).

### Plant material preparation for gene cloning

Grapevine leaf discs (14 mm diameter) from the sixth leaf counted from the apex were treated with 1% AlCl_3_ or UV for ROMT gene induction as described in Schmidlin *et al*. (2008). Alternatively, discs were also treated with 0.1 mm MeJA, 5 mm MBCD or 0.1 mm MeJA + 5 mm MBCD. Leaf discs were collected 24 h after treatment, immediately frozen in liquid nitrogen, and stored at −80 °C until RNA extraction.

*Vitis vinifera* cv. Gamay cell suspensions were elicited as previously described (Martinez‐Esteso *et al*., 2009, 2011a) for STS gene induction, using 50 mm MBCD and 0.1 mm MeJA. The cell suspension was incubated for 24 h with continuous rotary shaking (110 rpm) at 25 °C and under a 16 h light/8 h dark photoperiod. Cells were collected by filtration under gentle vacuum and stored at −80 °C until RNA extraction.

### RNA isolation and cloning of ROMT and STS cDNA

Total RNAs were isolated as described in Morante‐Carriel *et al*. (2014) from 0.5 g grapevine leaf discs of each treatment or 1 g grapevine elicited cells, and quantified using a Nanodrop ND‐1000 spectrophotometer (Thermo Scientific, Madrid, Spain). Residual genomic DNA was removed using DNase I RNase‐Free (Thermo Scientific, Madrid, Spain. First‐strand cDNA was synthesized from 1 μg of total RNA using a cDNA synthesis kit (RevertAid First Strand cDNA Synthesis Kit from ThermoScientific) according to the manufacturer's instructions. ROMT coding region (Acc. NM_001281115.1) was PCR amplified from leaf cDNA, and STS coding region (Acc. XM_002264953) was PCR amplified from elicited cell cDNA using specific primer (Table S1). The amplification reactions consisted of 1 cycle at 94 °C for 5 min and 30 cycles at 94 °C for 30 s, 54 °C for 30 s, 72 °C for 1 min, followed by an extension cycle of 10 min at 72 °C. Amplified DNA fragments were cloned into pGEM^®^‐T Easy (Promega, Madison, WI, USA) and the inserts sequenced.

### Construction of the binary vector and *Agrobacterium* transformation

The ROMT gene was cloned into pJCV52 vector (Laboratory of Plant Systems Biology; Ghent University, Belgium), and STS gene was cloned in pEarleyGate 103 (Earley *et al*., 2006) under the CaMV35S promoter using the Gateway cloning system (Invitrogen, Life Technologies, NY, USA). These constructs also contain the neomycin phosphotransferase (NPTII) selectable marker reporter gene. Briefly, pGEM^®^‐T Easy plasmid harbouring the target cDNA sequence was amplified by PCR using the proofreading Pfx DNA polymerase (Invitrogen) and the appropriate primers for ROMT and STS (Table S2). PCR products were inserted into the pENTR^™^/D‐TOPO^®^ vector (Invitrogen) as recommended by the manufacturer. Plasmids pENTR^™^/D‐TOPO^®^ containing cDNA insert in the correct orientation were selected by PCR, and the complete insert was subsequently sequenced. Correct inserts were transferred into the Gateway‐compatible vector pJCV52 or pEarleyGate 103 using an LR clonase reaction (Invitrogen) carried out according to the manufacturer's instructions.

Human liver cytochrome P450 CYP1B1 gene (Acc. BT019979.1) was obtained from commercial synthesis (GeneScript; Piscataway NJ, USA) and transferred into the Gateway‐compatible vector pK7WG2D (Laboratory of Plant Systems Biology; Ghent University, Belgium) under the CaMV35S promoter control. This construct also contain the neomycin phosphotransferase (NPTII) selectable marker reporter gene.

**Figure 9:**
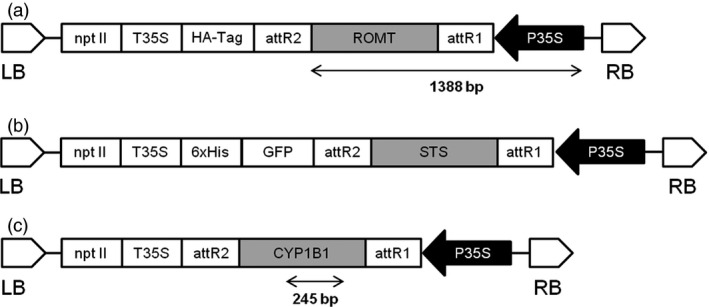
Schematic diagram of T‐DNA region of binary plasmids used for transformation experiments. P35S, CaMV 35S promoter; nptII, neomycin phosphotransferase under the control of the P35S promoter; GFP, green fluorescent protein; T35S, CaMV 35S terminator; STS, stilbene synthase; ROMT, resveratrol O‐methyltransferase; CYP1B1, Cytochrome P450; LB, left border; RB, right border. (a) pJCV52‐ROMT. (b) pEarleyGate‐STS. (c) pK7WG2D‐CYP1B1.

The binary vector pJCV52‐ROMT (Figure 9a) was transferred into chemically competent *A. tumefaciens* strain EHA105 (Hood *et al*., 1993) and the pEarleyGate‐STS (Figure 9b) and the pK7WG2D‐CYP1B1 (Figure 9c) to chemically competent *A. tumefaciens* strain C58C1 (pGV2260) (Koncz and Schell, 1986) by standard techniques (Sambrook *et al*., 1989).

### Transient expression in *Nicotiana benthamiana*

*A. tumefaciens* strain carrying binary vectors pJCV52‐ROMT; pK7WG2D‐CYP1B1 or pEarleyGate‐STS were used for transient expression in *Nicotiana* leaf by agroinfiltration (Schöb *et al*., 1997). The strains harbouring the binary plant vectors produced, pJCV52‐ROMT and pEarleyGate‐STS or pK7WG2D‐CYP1B1 and pEarleyGate‐STS, were co‐infiltrated with a strain containing the HC‐Pro silencing suppressor (Goytia *et al*., 2006) in a 1 : 1 : 2 ratio in leaves of *Nicotiana* plants. From 6 days after agro‐infiltration, stilbenes content was analysed.

### Stable transformation of grapevine cell culture

Transformation experiments were performed as described in Martínez‐Márquez *et al*. (2015) from 10 g *Vitis* callus. Then, the biomass was transferred onto solid Gamborg B5 medium containing 100 mg/L acetosyringone, and after 2 days of co‐culture, cells were transferred onto solid Gamborg B5 medium containing 250 mg/L cefotaxime and 60 mg/L paramomycin. Periodical subcultures of growing callus colonies were carried out at cefotaxime decreasing concentrations.

### DNA extraction, amplification and analysis

Genomic DNA was isolated from 150 to 300 mg of *Vitis* cells using E.Z.N.A. HP Plant DNA Mini Kit (OMEGA Bio‐tek, Norcross GA, USA), according to the manufacturer's instructions. The presence of the cassette P35S‐ROMT, CYP1B1 and the absence of virB genes in *Vitis* transgenic calli was assessed by PCR analysis, where specific primers were used for amplifying a 1388 bp fragment of the cassette P35S‐ROMT coding region, a 245 bp fragment of the CYP1B1 coding region and a 800 bp fragment of the virB coding region (Table S3) (Figure 9). The amplification reactions are as follows: 1 cycle at 95 °C for 5 min and 30 cycles at 94 °C for 1 min, 54 °C for 1 min, 72 °C for 1.30 min, followed by an extension cycle of 10 min at 72 °C. Plasmid DNA used in transformation served as a positive control, while DNA from nontransformed wild‐type *Vitis* cells was used as a negative control. PCR products were analysed by electrophoresis on 1% agarose gels.

### Protein extraction and Western Blot

Protein extracts from *Vitis* calli were prepared as described in Martínez‐Esteso *et al*. (2011b) from 5 g grapevine cells. After homogenization, the extract was centrifuged and the supernatant which containing native soluble protein fraction was stored at −80 °C for enzyme assays. One half of this supernatant was processed by adding an equal volume of Tris‐saturated phenol pH 7.5 (Applichem, Darmstadt, Germany). The precipitated and air‐dried proteins were solubilized in 1× SDS‐PAGE sample buffer. The protein concentration was determined by RC DC protein assay (BIO‐RAD, Madrid, Spain) based on the modified Lowry protein assay method (Raghupathi and Diwan, 1994).

Proteins (50 μg/lane) were resolved by SDS‐PAGE on 12.5% polyacrylamide gels and electrotransferred to Hybond‐P PVDF membranes (GE Healthcare, Madrid, Spain). Membranes were probed at 4 °C overnight with rabbit monoclonal anti‐HA‐Tag antibodies (Sigma) at a 1 : 1000 dilution to detect the HA‐ROMT fusion protein or with rabbit monoclonal antibodies to CYP1B1 (Thermo Scientific, Pierce, Madrid, Spain) at a 1 : 750 dilution. Thereafter, this was incubated at 25 °C for 1 h with horseradish peroxidase‐conjugated goat anti‐rabbit IgG (Pierce) at a 1 : 10000 dilution. Detection was performed by ECL using the Prime Western Blotting Detection Reagent SuperSignal West Dura system (GE Healthcare‐Amersham).

### ROMT enzyme assay

Soluble native protein (400 μg total proteins) was incubated in a final volume of 1 mL with 25 mm S‐(5′‐Adenosyl)‐l‐methionine chloride dihydrochloride (SAM) and 500 μm of *t*‐R in 0.1 m Tris, pH 7.5, containing 20% glycerol (v/v), 5 mm MgCl_2_ and 14 mm 2‐mercaptoethanol. Reaction products were analysed by LC in an Agilent 1100 series HPLC equipped with UV–vis and ESI‐MS/MS ion trap detectors.

### Elicitation of transformed grapevine cell cultures

Elicitation was carried out as described (Lijavetzky *et al*., 2008; Martinez‐Esteso *et al*., 2011a). Briefly, a weighted amount of filtered and washed cells was transferred to a 250 mL Erlenmeyer flask and suspended in sterile fresh standard medium containing elicitors (50 mm MBCD and 0.1 mm MeJA) at a final volume of 100 mL. The cell suspension was incubated with continuous rotary shaking (100 rpm) at 25 °C and under a 16 h light/8 h dark photoperiod from Gamay and continuous dark from Monastrell. Samples were taken at time intervals from elicited cultures of CYP1B1 and ROMT transgenic grapevine cell cultures. The spent medium and the cells were used for extracellular and intracellular extraction and analysis of piceatannol or pterostilbene content, respectively. Experiments were carried out in triplicate.

### Determination of stilbenoids

Extracellular stilbenes were extracted twice with 25% ethyl acetate (v/v), evaporated and resuspended in 80% methanol. For intracellular samples, 1 g of fresh cells was extracted with 4 mL of methanol overnight at 4 °C with continuous shaking and the extract was clarified by centrifugation at 14 000 ***g*** for 10 min. For agroinfiltration samples, 1 g of leaves was ground in liquid nitrogen and extracted with 8 mL of methanol overnight at 4 °C with continuous shaking and the extract was clarified by centrifugation at 14 000 ***g*** for 10 min. For stilbenoid determination, 10 μL of sample was analysed by LC in an Agilent 1100 series HPLC equipped with UV–vis and ESI‐MS/MS detectors (Lijavetzky *et al*., 2008). The sample injected into a Poroshell 120 EC‐C18 (4.6 × 100 mm 2.7 microns) (Agilent, Palo Alto, CA, USA) was eluted in a gradient of solvents A (0.05% TFA) and B (0.05% TFA in methanol:acetonitrile 60 : 40 v/v) at a flow rate of 1 mL/min. The gradient consisted of 0 min, 22.5% B; 4 min, 35% B; 8 min, 40% B; 14 min, 65% B; 19 min, 65% B; 21 min, 22.5% B; 23 min, 22.5% B. The *t*‐Resveratrol (*t*‐R), *t*‐Piceid (*t*‐Pc), *t*‐Piceatannol (*t*‐Pn) and *t*‐Pterostilbene (*t*‐Pt) standards were purchased from ChromaDex Inc. (Irvine, CA, USA). Calibration curves were made for quantification of these compounds in the samples obtained from the cell cultures.

## Supporting information


**Figure S1** MS/MS spectrum of pterostilbene (m/z 257) in transgenic lines of *Vitis vinifera* cv. Monastrell and cv. Gamay extracellular medium.
**Figure S2** MS/MS spectrum of pterostilbene (m/z 257) in transgenic lines of *Vitis vinifera* cv. Monastrell and cv. Gamay cell extracts.
**Figure S3** MS/MS spectrum of piceatannol (m/z 245) in transgenic lines of *Vitis vinifera* cv. Monastrell and cv. Gamay extracellular medium.
**Figure S4** MS/MS spectrum of piceatannol (m/z 245) in transgenic and wild‐type lines of *Vitis vinifera* cv. Monastrell and cv. Gamay cell extracts.
**Table S1** Gene specific primers.
**Table S2** Gene specific primers.
**Table S3** Gene specific primers.
