# Supplementary material for: Production of highly bioactive resveratrol analogues pterostilbene and piceatannol in metabolically engineered grapevine cell cultures
Source: Plant Biotechnol J. 2016 Mar 7;14(9):1813–25. doi: 10.1111/pbi.12539 (PMC5069453; doi:10.1111/pbi.12539)
Supplement: Supplementary file 1 — Figure S1 MS/MS spectrum of pterostilbene (m/z 257) in transgenic lines of Vitis vinifera cv. Monastrell and cv. Gamay extracellular medium. Figure S2 MS/MS spectrum of pterostilbene (m/z 257) in transgenic lines of Vitis vinifera cv. Monastrell and cv. Gamay cell extracts. Figure S3 MS/MS spectrum of piceatannol (m/z 245) in transgenic lines of Vitis vinifera cv. Monastrell and cv. Gamay extracellular medium. Figure S4 MS/MS spectrum of piceatannol (m/z 245) in transgenic and wild‐type lines of Vitis vinifera cv. Monastrell and cv. Gamay cell extracts. Table S1 Gene specific primers. Table S2 Gene specific primers. Table S3 Gene specific primers. [file PBI-14-1813-s001.docx]

**Supporting information:**

**Production of highly bioactive resveratrol analogues pterostilbene and piceatannol in metabolically engineered grapevine cell cultures**

**Martínez- Márquez A. et al.**

**Table S1: Gene specific primers**

|  | **Upstream primer** | **Downstream primer** |
| --- | --- | --- |
| **ROMT** | 5´-ATGGATTTGGCAAACGCTGTGATATCAGCTGA-3´ | 5´-TCAAGGATAAACCTCAATGAGGGACCTCAAACC-3´ |
| **STS** | 5´- ATGGCGTCTGTGGACGAAATTAGA-3´ | 5´-TTAGTTTGAATCCATACAAATGCT-3’ |

**Table S2: Gene specific primers**

|  | **Upstream primer** | **Downstream primer** |
| --- | --- | --- |
| **ROMT** | 5´-CACCATGGATTTGGCAACG-3´ | 5´-AGGATAAAC CTCAATAG-3´ |
| **STS** | 5´-CACCATGGCGTCTGTGGACGAAATTAGA-3´ | 5´-GTTTGAATCCATACAAATGC-3’ |

**Table S3: Gene specific primers**

|  | **Upstream primer** | **Downstream primer** |
| --- | --- | --- |
| **P35S-ROMT** | 5´-GCACCTACAAATGCCATCA-3´ | 5´-AGGATAAACCTCAATGAGGGAC-3´ |
| **CYP1B1** | 5´-CCTATGTCCTGGCCTTCCTT-3´ | 5´-ACTCTGCTGGTCAGGTCCTT-3´ |
| **virB** | 5´-TCGGGCACCGTCAGCTTGACG-3´ | 5´-GTTAAGAAGATCGCCTATTGT-3´ |

**
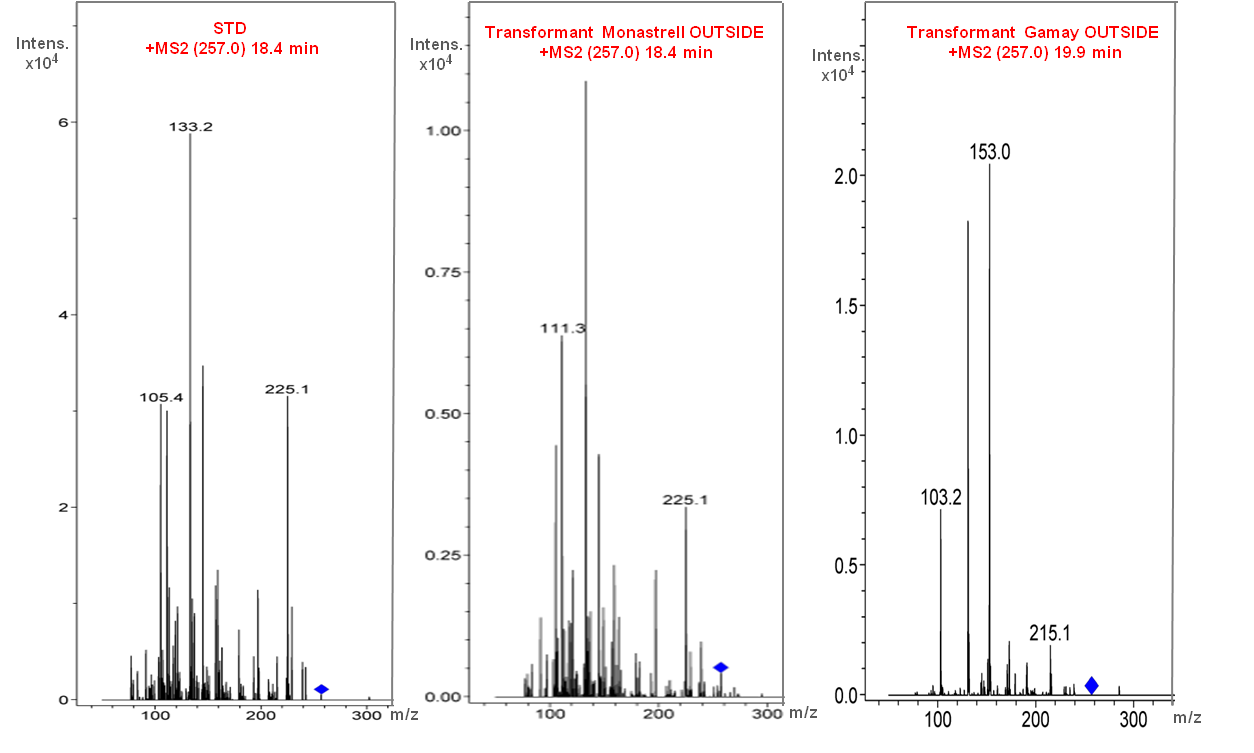
**

**Figure S1:** MS/MS spectrum of pterostilbene (m/z 257) in transgenic lines of *Vitis vinifera* cv. Monastrell and cv. Gamay extracellular medium.


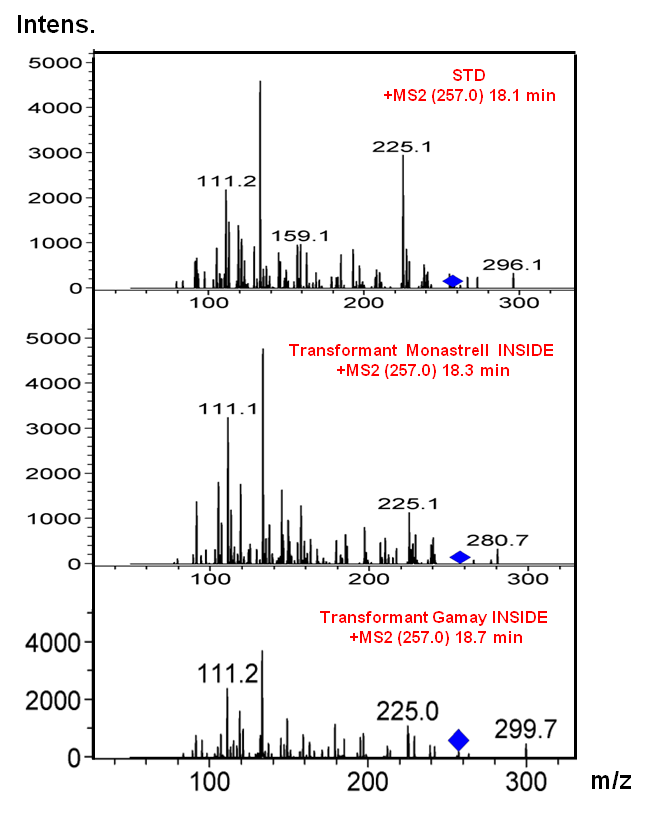


**Figure S2**: MS/MS spectrum of pterostilbene (m/z 257) in transgenic lines of *Vitis vinifera* cv. Monastrell and cv. Gamay cell extracts.


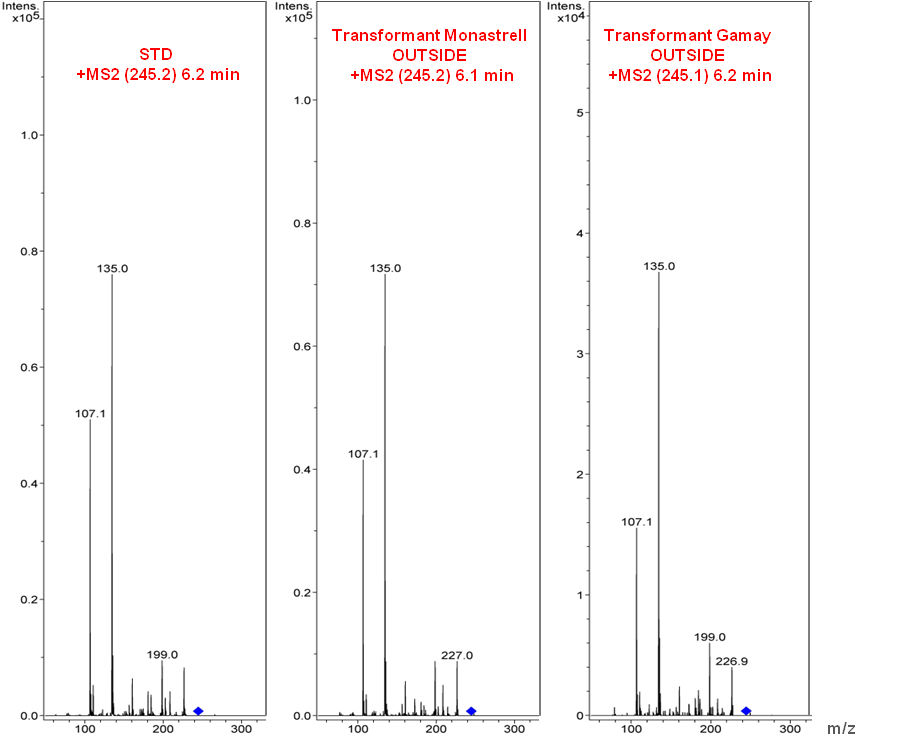


**Figure S3:** MS/MS spectrum of piceatannol (m/z 245) in transgenic lines of *Vitis vinifera* cv. Monastrell and cv. Gamay extracellular medium.


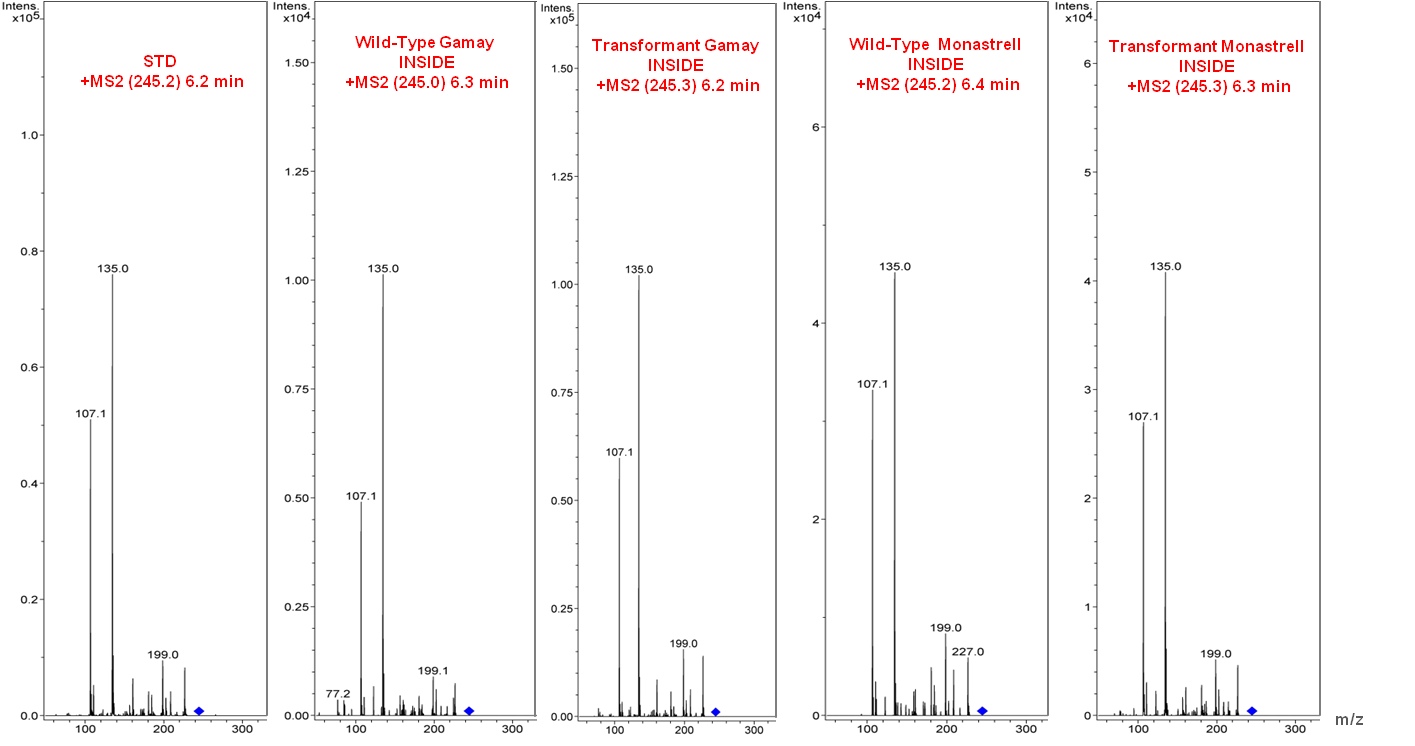


**Figure S4**: MS/MS spectrum of piceatannol (m/z 245) in transgenic and wild-type lines of *Vitis vinifera* cv. Monastrell and cv. Gamay cell extracts.
